# Biodegradation of endocrine disruptor dibutyl phthalate (DBP) by a newly isolated *Methylobacillus* sp. V29b and the DBP degradation pathway

**DOI:** 10.1007/s13205-016-0524-5

**Published:** 2016-09-21

**Authors:** Vinay Kumar, S. S. Maitra

**Affiliations:** Lab No. 117, School of Biotechnology, Jawaharlal Nehru University, New Delhi, 110067 India

**Keywords:** Endocrine disruptor, Degradation kinetics, Stoichiometry, Gene identification, Phthalate ester degradation pathway

## Abstract

Bacteria of the genus *Methylobacillus* are methanotrophs, a metabolic feature that is widespread in the phylum Proteobacteria. The study demonstrates the isolation and characterization of a newly isolated *Methylobacillus* sp. V29b. which grows on methanol, protocatechuate, monobutyl phthalate, dibutyl phthalate, diethyl phthalate, benzyl butyl phthalate, dioctyl phthalate and diisodecyl phthalate. *Methylobacillus* sp. V29b was characterized with scanning electron microscopy, transmission electron microscopy, Gram staining, antibiotics sensitivity tests and biochemical characterization. It degrades 70 % of the initial DBP in minimal salt medium and 65 % of the initial DBP in samples contaminated with DBP. DBP biodegradation kinetics was explained by the Monod growth inhibition model. Values for maximum specific growth rate (*µ*_max_) and half-velocity constant (*K*_s_) are 0.07 h^−1^ and 998.2 mg/l, respectively. Stoichiometry for DBP degradation was calculated for *Methylobacillus* sp. V29b. Four metabolic intermediates, dibutyl phthalate (DBP), monobutyl phthalate, phthalic acid and pyrocatechol, were identified. Based on the metabolic intermediates identified, a chemical pathway for DBP degradation was proposed. Six genes for phthalic acid degradation were identified from the genome of *Methylobacillus* sp. V29b.

## Introduction

Phthalic acid esters (PAEs) are a class of compounds widely used as plasticizers to provide mechanical strength and flexibility to the resins (Cartwright et al. 2000; Staples et al. 1997). They are ingredients of paints, adhesives, house-building materials, defoaming agents, PVC pipes, food packing materials, toys, plastics, solubilizers of cosmetic products, medical devices, photography films, textile fabrics, pesticide carriers, lubricating oils and are used in aerospace technology (Gesler 1973; Gross and Colony 1973; Hauser et al. 2007; Ito et al. 2005; Krauskopf 1973; Marcel 1973; Teil et al. 2006; Tepper 1973; Wang et al. 1995b; Wilkinson and Lamb 1999). They have low solubility in water; therefore, they are stable in the environment over a longer period of time (Huang et al. 2008; Vikelsøe et al. 2002; Wang et al. 2008; Yuan et al. 2002). They have been detected in various environments including landfill leachates (Schwarzbauer et al. 2002; Zheng et al. 2007), air (Wensing et al. 2005), soils, sediments, and natural waters (Staples et al. 1997). PAEs have been classified as top priority toxicants by the European Union, China National Environmental Monitoring Center and US Environment Protection Agency (Chen et al. 2007; Council of the European Union 1993). PAEs are responsible for carcinogenicity and endocrine disruption (Colborn et al. 1993; David et al. 1999; Jobling et al. 1995; Piersma et al. 2000). They can elicit cellular estrogenic responses and suppress calcium signaling (Kaun-Yu et al. 2004; Lovekamp-Swan and Davis 2003; Moore 2000). They are responsible for hypospadias, cryptorchidism and malformation of the reproductive tract in mice (Fisher 2004; Gray et al. 2000; Jaeger and Rubin 1970; Li et al. 1998; Zhu et al. 2006). They are known for irritation of eyes, skin, respiratory tract, blurred vision and induce stone formation in the bladder (Dai et al. 2005a, b; Wang and Gu 2006). Dibutyl phthalate (DBP) is the most widely used plasticizer and has been detected in different environments (Eaton 2001; Feiler 1980; Hashizume et al. 2002; Keith and Telliard 1979; Xu et al. 2005).

The natural processes of degradation such as hydrolysis and photodecomposition are not efficient in the degradation of these pollutants (Lu et al. 2009). Therefore, microbial degradation is the major route for their degradation (Staples et al. 1997; Vamsee-Krishna et al. 2006; Vamsee-Krishna and Phale 2008). Bacteria having potential to degrade PAEs have been isolated from various environments including mangrove sediments, activated sludge and wastewater (Liang et al. 2008). Aerobic degradation of PAEs is much more efficient as compared to anaerobic degradation (Cheung et al. 2007; Fang et al. 2010). Sequential hydrolysis of PAEs has been demonstrated by a few researchers (Engelhardt and Wallnöfer 1978; Jiao et al. 2013; Staples et al. 1997).

Despite research on degradation of PAEs by various researchers, these studies lack in perspectives such as efficient DBP degradation at higher concentrations, elucidation of DBP degradation pathway, kinetics of DBP degradation and identification of genes responsible for PAEs degradation. Extensive research in the above aspects is required to remove these pollutants from the environment. The aim of the study was isolation, characterization and identification of efficient DBP-degrading bacterial strain from municipal solid waste (MSW) leachate and to examine the degradation potential of the isolate toward degradation of DBP in both minimal media and in PAEs-contaminated samples collected from the landfill site.

## Materials and methods

### Chemicals

HPLC-grade monobutyl phthalate, diisodecyl phthalate, dioctyl phthalate, protocatechuate, benzyl butyl phthalate, diethyl phthalate and dibutyl phthalate were purchased from Sigma-Aldrich (USA) and used as substrate for growth of the bacteria. HPLC-grade acetonitrile purchased from Sigma-Aldrich (USA) was used as solvent in the analysis of DBP.

### Isolation and characterization of DBP-degrading bacteria

Municipal solid waste (MSW) leachate samples were collected from a municipal solid waste landfill site at Ghazipur, New Delhi, India. The location co-ordinates of Ghazipur landfill site are 28° 37′ 22.4″N and 77° 19′ 25.7″E. The physical parameters of the site are: pH 8.4, TDS 29,700, COD 31,600, Fe 9.81 and Cl 1174.2. After collection, the samples were stored at 4 °C. The MSW leachate was inoculated in minimal salt medium (MSM) supplemented with DBP [DBP emulsified with 0.1 % (vol/vol) Tween 80] as the sole carbon and energy source. MSM was prepared by dissolving 3.5 g K_2_HPO_4_, 1.5 g KH_2_PO_4_, 0.27 g MgSO_4_, 1 g NH_4_Cl, 0.03 g Fe_2_(SO_4_)_3_·7H_2_O and 0.03 g CaCl_2_ in 1 L distilled water. The pH of MSM was adjusted to 6.8 and sterilized by autoclaving at 121 °C and 15 psi for 20 min. A separate iron sulfate and magnesium sulfate solution was prepared, filter sterilized with 0.22 µm membrane filter and added to MSM to avoid precipitate formation (Vega and Bastide 2003). For isolation of DBP-degrading bacteria, 1 ml MSW leachate was inoculated to 100 ml MSM supplemented with 10 mg/l of DBP and incubated at 30 °C and 180 rpm. The amount of DBP was increased in subsequent transfer cultures. Culture from the flask was streaked on MSM–agar plates to obtain a pure culture. A colony numbered 29 was able to grow in the presence of DBP and it was designated as strain 29D. Size and cell morphology were observed using scanning electron microscopy (SEM). Internal features of the bacteria were observed using transmission electron microscopy (TEM). SEM and TEM of strain 29D were performed at the Advanced Instrumentation Research Facility (AIRF), Jawaharlal Nehru University, New Delhi. For electron microscopy, bacterial cells were fixed in 3 % glutaraldehyde at room temperature for 3 h. After fixation, bacterial cells were washed with 0.1 M phosphate buffer thrice for 10 min. Post-fixation was performed in 1–2 % osmium tetroxide solution followed by dehydration with increasing concentrations of ethanol in water solution (Smith 1955). Characterization of strain 29D was performed using biochemical tests (Vos et al. 2011). Gram staining reaction was performed using Gram-stain kit from Himedia Lifesciences. Determination of catalase activity was performed by assessment of bubble production in 3 % (v/v) H_2_O_2_. Determination of oxidase activity was performed using 1 % (w/v) tetramethyl-p-phenylenediamine from Himedia lifesciences. Hydrolysis of starch was determined by growing bacteria on MSM plates containing 0.2 % (w/v) starch. Susceptibility to antibiotics was determined by spreading the bacterial suspension on MSM plates amended with 10–100 µg/ml of antibiotic tested. To assess the capability of strain 29D to cleave the benzene ring, Rothera’s test was performed. For the test, 5 ml of bacterial culture was saturated with solid ammonium sulfate and mixed with a few drops of 2 % sodium nitroprusside solution and liquor ammonia. The mixture was left for 15 min. A bluish-purple ring indicates the presence of the ketone bodies in it (Rothera 1908).

### 16S-rRNA gene amplification, sequencing and phylogenetic analysis

Identification of strain 29D was performed by 16S-rRNA gene identification. Genomic DNA of the strain 29D was isolated using Fast DNA^**®**^ SPIN Kit for soil from MP Bio. The 16S-rRNA gene amplification was performed with bacterial universal primers 27F and 1492R (Weisburg et al. 1991). For PCR, a 50 µl reaction was used containing 25 µl PCR master mix (Thermo Scientific), 2 µl forward primer, 2 µl reverse primer, 23 µl sterile water and 1 µl genomic DNA. Time programming used for the thermo cycler (Applied Biosystems Gene Amp PCR system 9700) was 10 min at 95 °C, 35 cycles, 60 s at 95 °C, 90 s at 54 °C, and 60 s at 72 °C and 5 min at 72 °C. The amplified PCR product was gel purified with HiYield™ PCR DNA Mini Kit from Real Genomics™ (Ref catalog no. YDF100). Sequencing of the purified product was performed at the DNA sequencing facility UDSC, University of Delhi, New Delhi, India. Sequencing was performed using dideoxy termination method with bacterial universal primers: 27F (5′-AGAGTTTGATCCTGGCTCAG-3′) and 1492R (5′-GGCTACCTTGTTACGACTT-3′). The obtained sequences were combined with BioEdit program. The resultant 16S-rRNA sequence was compared with the representative sequences of bacterial organisms from GenBank and aligned using CLUSTAL W (Thompson et al. 1994). The 16S-rRNA sequence was submitted to NCBI. Phylogenetic analyses were carried out with maximum composite likelihood method (Felsenstein 1981) using MEGA version 5 (Tamura et al. 2011).

### Substrate utilization

Substrate utilization of strain 29D was performed to examine its ability to grow on different substrates. Six substrates were chosen including methanol, diethyl phthalate (DEP), dioctyl phthalate (DOP), monobutyl phthalate (MBP), diisodecyl phthalate (DIDP), benzyl butyl phthalate (BBP) and protocatechuic acid (PC). Substrate concentration was 2000 mg/l. Growth of strain 29D was measured with Perkin Elmer Lambda 25 UV/vis spectrophotometer at 600 nm.

### Kinetics and stoichiometry of DBP degradation by strain 29D

For degradation studies, strain 29D was inoculated in MSM and PAEs-contaminated samples and flasks were incubated at 30 °C and 180 revolution per minute in an incubator. Samples for residual DBP analysis and metabolic intermediates identification were collected every 24 h. Collected samples were extracted with ethyl acetate in 1:1 ratio and residues were dissolved in methanol. The obtained extract was filtered through a 0.22 µm membrane filter and the filtrate was transferred to an autosampler vials for HPLC and gas chromatography analysis (Jin et al. 2010). For HPLC analysis of the extracts, 20 µl of the filtrate was injected to the Shimadzu HPLC system. Analysis of the samples was performed using Ascentis^®^ C 18 column, 5 µm, 25 cm × 4.6 cm from Sigma-Aldrich. A gradient program having two mobile phases, a water/acetonitrile (15:85) v/v and B 100 % acetonitrile, was used. Time programing was: 0–3 min a 100 % A, 6.5–19.5 min 100 % B. A total flow rate of 0.6 ml/min was maintained. Run time of the samples was 45 min. DBP was detected using a UV detector at 225 nm (Thuren 1986). Residual DBP in the samples was quantified by preparing a standard curve for DBP (Park et al. 2016). Metabolic intermediates for DBP degradation were identified by the GC–MS system at Advanced Instrumentation Research Facility, Jawaharlal Nehru University, New Delhi, India, with column temperature of 100 °C, injection temperature 250 °C and total flow 16.3 ml/min. For calculation of biomass in terms of dry weight, bacterial cells were harvested and filtered with 0.45 µm membrane filter and dried in an oven at 100 °C. The dried biomass was measured with a weighing balance (Bratbak and Dundas 1984).

### Identification of PAEs-degrading genes

Genes responsible for phthalate esters degradation from the strain 29D were identified by PCR. Primers known for PAEs degradation were synthesized and amplified (Table 2). The programing used for the PCR thermocycler was: 5 min at 95 °C, 30 cycles of 30 s at 95 °C, 30 s at Tm of corresponding primers, 90 s at 72 °C and final extension for 7 min at 72 °C (Han 2008). Gel-purified amplification products were sequenced at the DNA sequencing facility UDSC, University of Delhi, New Delhi, India. Sequencing of the amplicons was performed by specific primers for each gene and amplicons were cloned in M13 vector.

**Tabel 1:** Growth of *Methylobacillus* sp. V29b in different substrates

Strain name	*Methylobacillus* sp. V29b
Methanol	+
PC	+++
MBP	+++
DEP	++
BBP	+
DOP	+
DIDP	+

*PC* pyrocatechol, *MBP* monobutyl phthalate, *DEP* diethyl phthalate, *BBP* benzyl butyl phthalate, *DOP* dioctyl phthalate, *DIDP* diisodecyl phthalate

### Statistical analysis

Statistical analysis of DBP degradation was performed by *F* test and one-way ANOVA with three replicates data using data analysis tool pack in Microsoft excel. For analysis, the hypothesis was made at the 5 % level of significance to calculate *P* and *F* values.

## Results and discussions

### Isolation, characterization and identification of the bacteria

**Fig. 1:**
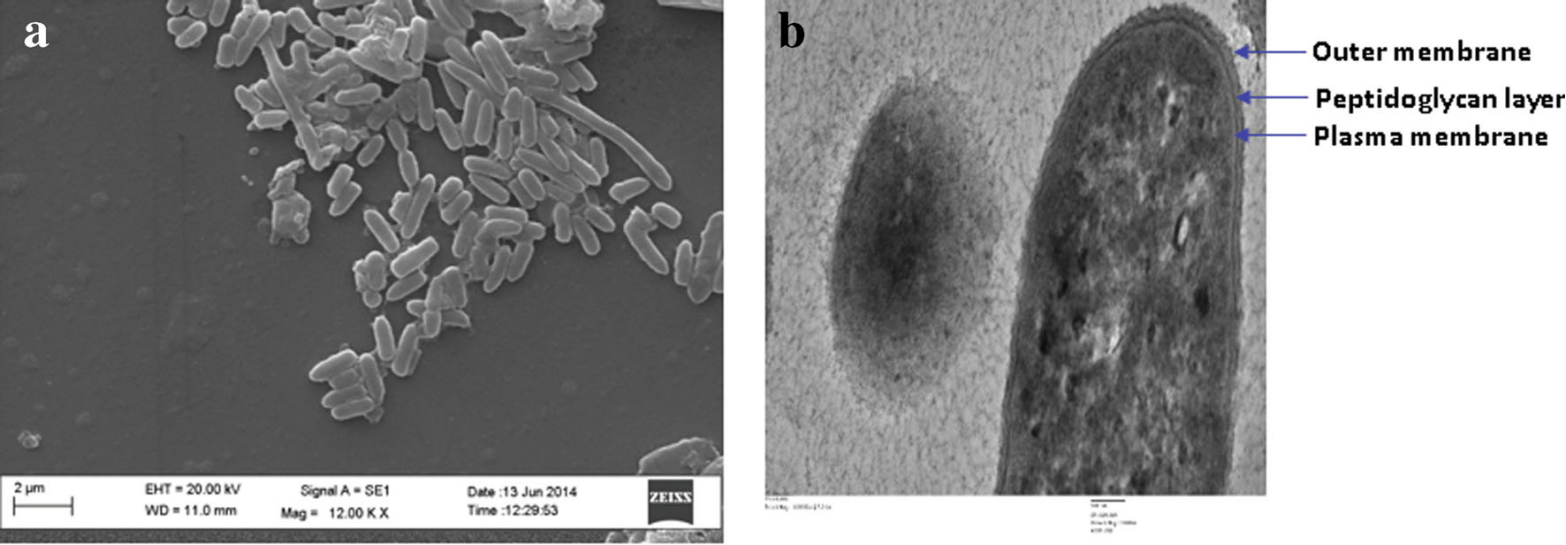
**a** Scanning electron micrograph of strain 29D. *Scale bar* 2 µm. **b** Transmission electron micrograph of strain 29D. *Scale bar* 100 nm

Cells of the strain 29D were rod shaped, Gram negative, aerobic and without flagella, forming round and creamy colonies on agar plates. The cells grow on MSM with 1 % methanol. Strain 29 D was catalase and oxidase positive which is the characteristic of bacteria form of the genus *Methylobacillus* (Doronina et al. 2004; Urakami and Komagata 1986; Anthony 1982; Lidstrom^ 2006^). Negative results were obtained for nitrate production, urease activity, H_2_S production, ammonia production, methyl red, Voges–Proskauer and starch hydrolysis. The strain was positive for indole production, lysine utilization and *β*-galactosidase activity. It was resistant to streptomycin (100 µg ml^−1^ and susceptible to ampicillin (10 µg ml^−1)^, penicillin (10 µg ml^−1^, kanamycin (10 µg ml^−1)^, tetracycline (10 µg ml^−1^), and chloramphenicol (10 µg ml^−1)^. SEM at 20 KX revealed that strain 29D was rod shaped, smooth, without flagella with length ~2 µm and width ~0.2 µm. TEM of strain 29D at 20 KX revealed the presence of the outer membrane, peptidoglycan layer and plasma membrane (Fig. 1).

**Fig. 2:**
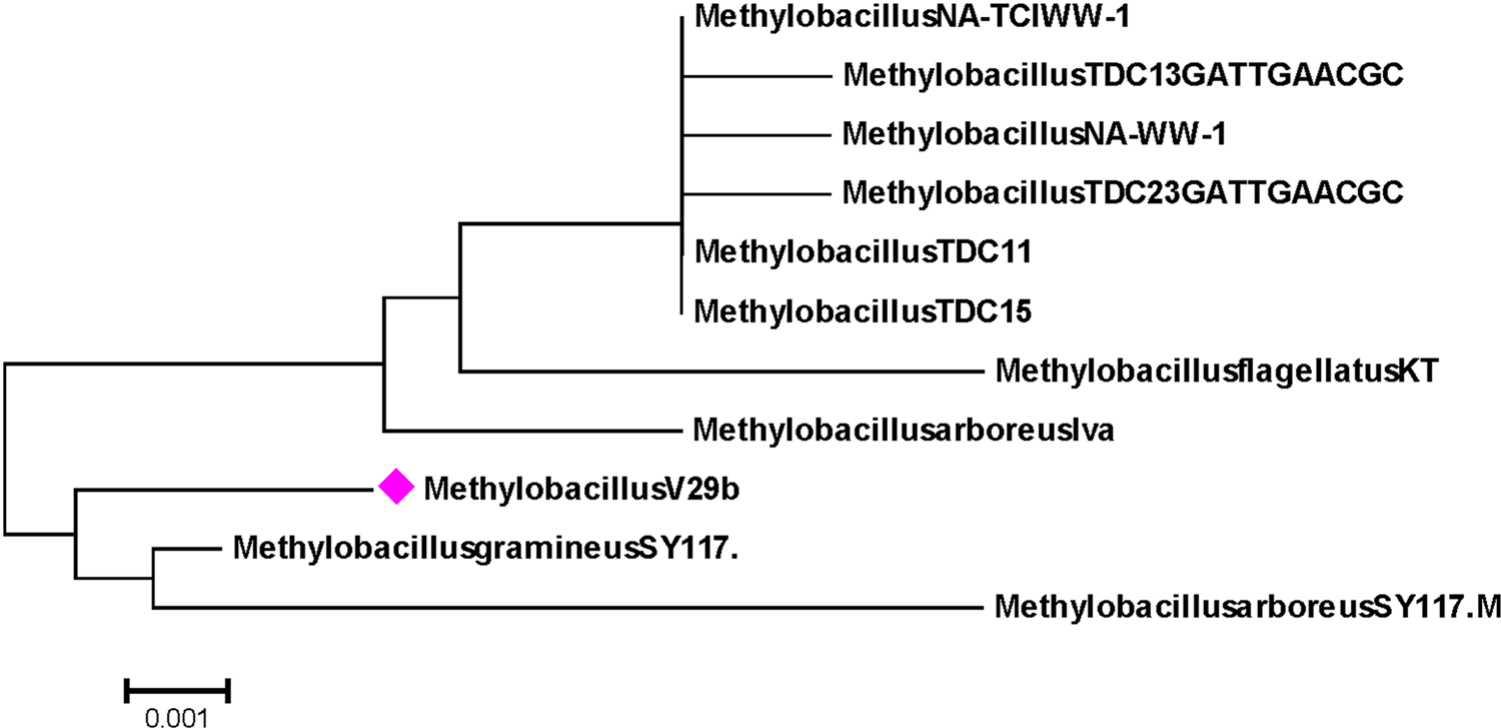
Phylogenetic tree of *Methylobacillus* sp. V29b. The evolutionary history was inferred using the UPGMA method. The optimal tree with the sum of branch length = 0.05032880 is shown. The percentage of replicate trees in which the associated taxa clustered together in the bootstrap test (500 replicates) are shown *above the branches*. The tree is drawn to *scale*, with branch lengths (*below the branches*) in the same units as those of the evolutionary distances used to infer the phylogenetic tree. The evolutionary distances were computed using the maximum composite likelihood method and are in the units of the number of base substitutions per site. Codon positions included were 1st + 2nd + 3rd + noncoding. All positions containing gaps and missing data were eliminated from the dataset (Complete deletion option). There were a total of 1281 positions in the final dataset. Phylogenetic analyses were conducted in MEGA5. The values such as 0.002 and 0.004 denote the evolutionary distance between different species and values such as 99 and 100 denote the similarities between different species

On comparison with the 16S-rRNA gene sequence of strain 29D, it found maximum identity with *Methylobacillus arboreus* clone SY117 (Accession No. KM041246.1). The strain 29D was designated as *Methylobacillus* sp. V29b and the obtained sequence was submitted to NCBI accession No. KM219114. The phylogenetic relationship of *Methylobacillus* sp. V29b is presented in Fig. 2.

### DBP biodegradation by *Methylobacillus* sp. V29b in MSM and contaminated samples

**Fig. 3:**
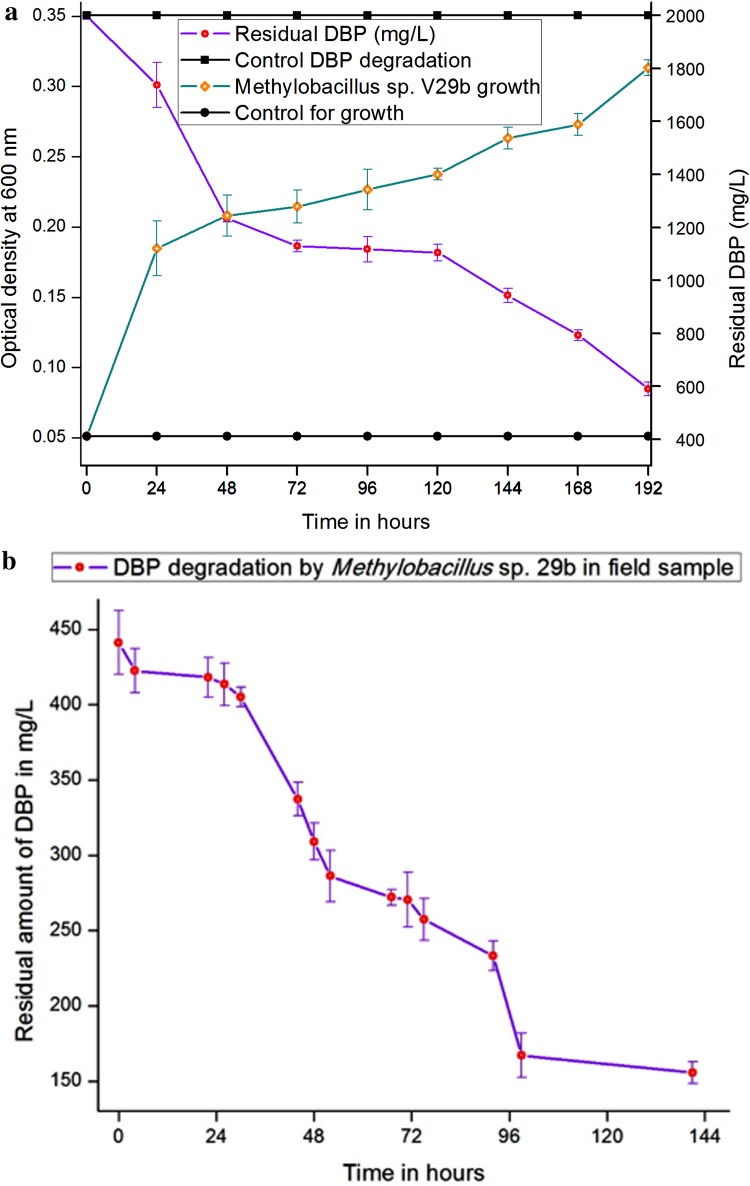
**a** Degradation of DBP by *Methylobacillus* sp. V29b in MSM. **b** Degradation of DBP by *Methylobacillus* sp. V29b in the sample contaminated with DBP

Biodegradation experiments were conducted by growing *Methylobacillus* sp. V29b in MSM supplemented with DBP at 2000 mg/l. To quantify the residual DBP in the bacterial culture, samples were collected every 24 h and quantified using HPLC. Figure 3a presents the relationship between the growth of *Methylobacillus* sp. V29b and DBP degradation. Figure 3b presents the degradation of DBP in the PAEs-contaminated sample from a landfill site at Ghazipur, New Delhi, India. The amount of DBP quantified in contaminated samples was 441 mg/l. From Fig. 3a, it was observed that from the initial DBP concentration of 1997 mg/l, *Methylobacillus* sp. V29b degraded half DBP in 120 h and only 590.40 mg/l DBP was left after 192 h. Therefore, 70.5 % of DBP was degraded in 192 h. From Fig. 3b, it may be observed that the *Methylobacillus* sp. V29b degraded half of the initial amount DBP in 96 h and 64.5 % the initial amount of DBP in 144 h.

### Biodegradation kinetics and DBP degradation stoichiometry

**Fig. 4:**
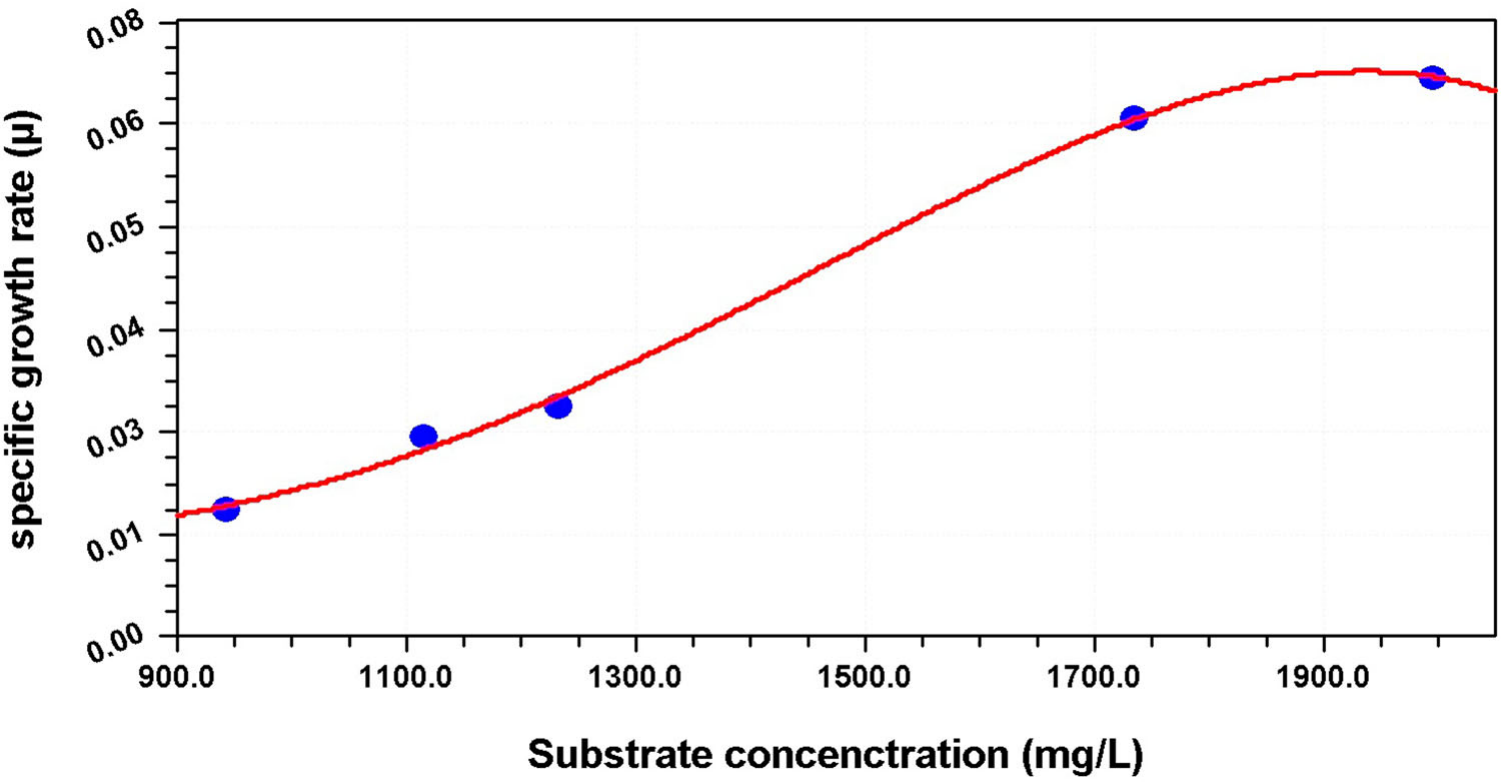
DBP degradation kinetics for *Methylobacillus sp. V29b*

Researchers have reported degradation kinetics using various models. Degradation kinetics of organic pollutants was explained by first-order equations (Lu et al. 2009; Xu et al. 2005; Zeng et al. 2004). A second-order equation was also reported for degradation of phthalate esters by algae *Chlorella pyrenoidosa* (Yan et al. 1995). A modified Gompertz model was used to describe the effect of initial DBP concentration on DBP biodegradation by *Gordonia* sp. QH-11(Jin et al. 2012). Haldane substrate inhibition model was used to explain DBP degradation in a continuous culture system (Wang et al. 1998). DBP biodegradation kinetics by *Methylobacillus* sp. V29b was explained by drawing a plot of specific growth rate (*µ*) and DBP concentration (*S*_av_) (Fig. 4). It was observed that as the concentration of DBP was increased, there was increase in specific growth rate, but when DBP concentration reached 1900 mg/l, there was a decline in the specific growth rate. This behavior was explained by the growth inhibition model. Equation (1) represents the Monod model for growth inhibition. The calculated maximum specific growth (*µ*_max_) and half-velocity constant (*K*_s_) were: 0.07 h^−1^ and 998.2 mg/l, respectively.

Monod model:

1 $$\mu = \frac{{\mu_{\hbox{max} } \times S}}{{(K_{\text{s}} + S)}},$$

where *μ* is the specific growth rate of the microorganism, *μ*_max_ the maximum specific growth rate of the microorganism, *S* the concentration of the limiting substrate for growth and *K*_s_ the half-velocity constant.

Values of the coefficients:

*µ*_max_ = 0.07 h^−1,^

*K*_s_ = 998.2 mg/l,

Yield = 0.43.

Stoichiometry for DBP utilization and biomass formation (Shuler and Kargi 2002) is presented in Eq. (2):

2 $$\begin{aligned} 50{\text{C}}_{ 1 6} {\text{H}}_{ 2 2} {\text{O}}_{ 4} + {\text{ 893O}}_{ 2} + { 2}0{\text{NH}}_{ 3} \to 20{\text{C}}_{ 5} {\text{H}}_{ 7} {\text{O}}_{ 2} {\text{N }} + {\text{ 546H}}_{ 2} {\text{O }} + { 7}00{\text{CO}}_{ 2} . \hfill \\ \quad \quad \quad {\text{DBP}}\quad \quad \quad \quad \quad \quad \quad \quad \quad \quad \quad \quad \quad {\text{Biomass}} \hfill \\ \end{aligned}$$

### Identification of metabolic intermediates

**Fig. 5:**
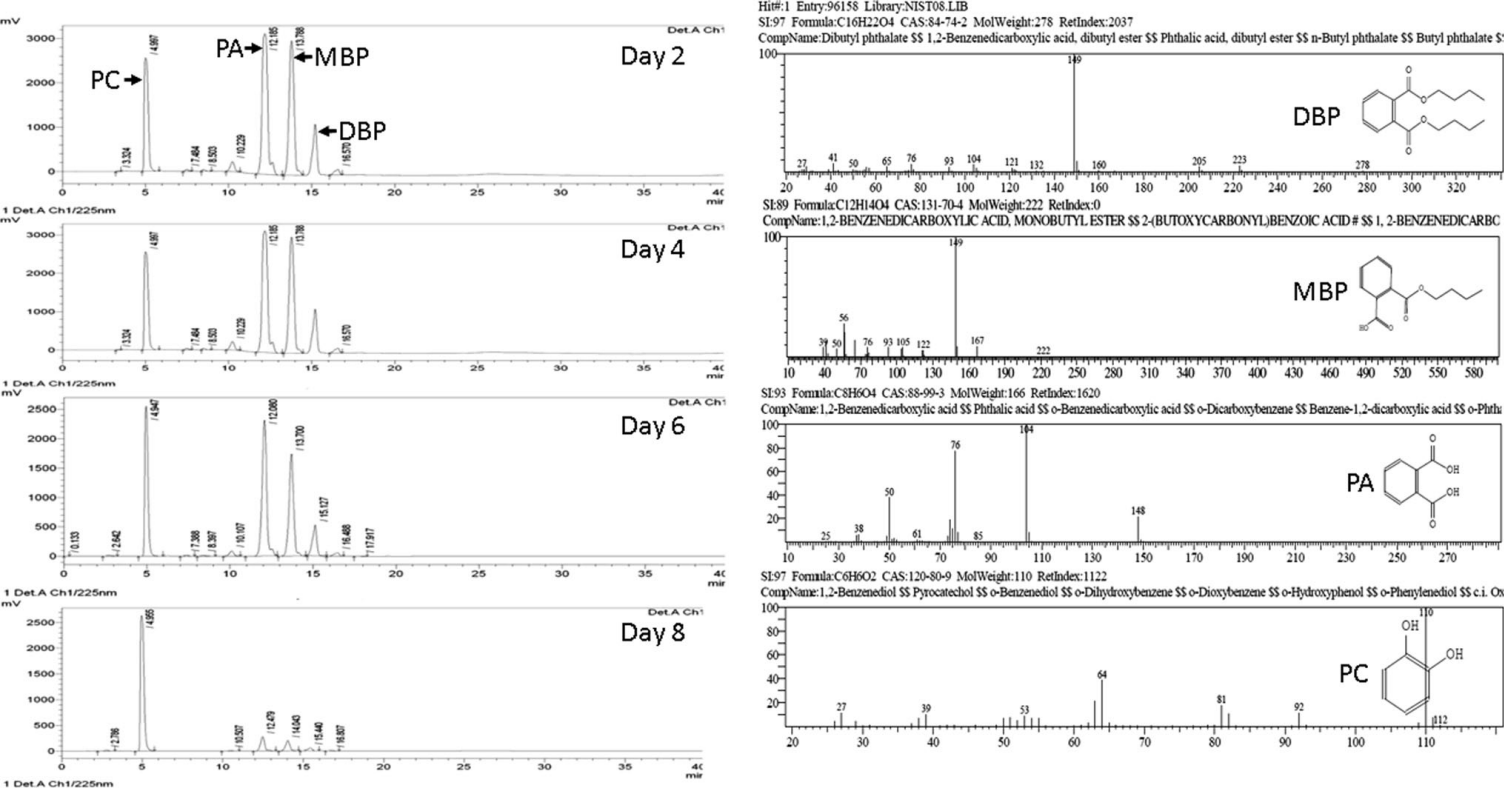
DBP degradation metabolic intermediates identified by GC–MS. **a** HPLC chromatogram of the metabolic intermediates. **b** Structure and *m*/*z* of the identified metabolic intermediates. *DBP* dibutyl phthalate, *MBP* monobutyl phthalate, *PA* phthalic acid, *PC* pyrocatechol

Determination of the metabolic intermediates for DBP degradation was performed by analysis of the GC–MS results. Four metabolic intermediates dibutyl phthalate (DBP), monobutyl phthalate (MBP), phthalic acid (PA), and pyrocatechol (PC) were detected during DBP degradation by comparing the mass spectrum at a particular retention time with published mass spectra from the National Institute of Standards and Technology (NIST) database. Figure 5 presents the HPLC peaks and structure of the identified metabolic intermediates from GC–MS. It was observed that with time the length of the DBP peak was decreased, while the peak lengths of the MBP and PA was increased and on day 8 the peak length was highest for pyrocatechol (PC). The identified intermediates suggest that DBP was converted to MBP, which was converted to PA. The final product of the reaction was PC. The positive result for Rothera’s reaction confirms the benzene ring cleavage.

**Fig. 6:**
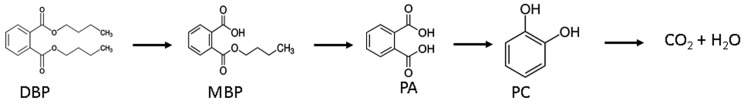
A proposed biochemical pathway for DBP degradation by *Methylobacillus* sp. V29b*. DBP* dibutyl phthalate, *MBP* monobutyl phthalate, *PA* phthalic acid, *PC* pyrocatechol

Few studies reported that PAEs degradation was mediated by a pathway where they are first converted to its monoester by esterase and then to phthalic acid (PA). PA is then transformed to carbon dioxide and water via an intermediate known as protocatechuate (Benckiser and Ottow 1982; Eaton and Ribbons 1982; Engelhardt and Wallnöfer 1978; Wang et al. 1995a, 2003a; Xu et al. 2005, 2007, 2006). Sometimes, two ester linkages in PAEs are cleaved by two different bacteria (Cartwright et al. 2000; Li et al. 2005; Li and Gu 2007). Some studies reported the formation of phthalic acid from DBP mediated by intermediate, monobutyl phthalate (MBP) (Benckiser and Ottow 1982; Gu et al. 2004; Wang et al. 2004; Xu et al. 2005). Based on the reported studies, the identified metabolic intermediates and Rothera’s test, a pathway for DBP degradation by *Methylobacillus* sp. V29b was proposed in Fig. 6. A similar pathway for dibutyl phthalate degradation in landfill bioreactor was reported for *Enterobacter* sp. T5 isolated from municipal solid waste (Fang et al. 2010). The study described the appearance of two major transient metabolites including phthalic acid (PA) and monobutyl phthalate (MBP). Pathway described for DBP degradation by *Methylobacillus* sp. V29b is the extension of the pathway proposed for DBP degradation by *Enterobacter* sp. T5. In *Methylobacillus* sp. V29b DBP, degradation of one more metabolic intermediate pyrocatechol was identified as the final aromatic intermediate which fills the gap between PA to carbon dioxide conversion.

### Identification of phthalates-degrading genes

*Methylobacillus* sp. V29b was able to grow on substrates protocatechuate, monobutyl phthalate, diethyl phthalate, benzyl butyl phthalate, dioctyl phthalate and dodecyl phthalate (Table 1). The growth of *Methylobacillus* sp. V29b decreases as the length and complexity of hydrocarbon attached to the phthalate ring increases (Wang et al. 2003b). Therefore, good growth was observed in PC, MBP and DEP. To examine the possibility for degradation of different phthalate esters by *Methylobacillus* sp. V29b and explore the PAEs degradation pathway, PAEs-degrading genes were amplified. Table 2 presents the list of primers selected and synthesized for gene amplification. Table 3 presents the genes amplified from *Methylobacillus* sp. V29b genome. Gene sequences for primers FEH, HFDH, FOXG, FOXGS and FDK were obtained from Arthrobacter sp. 68b growing on phthalic acid as the sole carbon source (Stanislauskienė et al. 2011). Gene sequence for the primer transporter ATPase was obtained from alkylbenzene-degrading *Rhodococcus* sp. strain DK17 which utilizes phthalate and terephthalate as growth substrates ((Choi et al. 2005). Gene sequences for the primers oph-A1, -A2, -B, -C, -D, -H, -R, Tph-A2, -A3, and -B were obtained from the bacteria-degrading phthalate, isophthalate and terephthalate (Han 2008).

**Table 2:** Primers used for identification of PAEs-degrading genes

Primer name	Gene name	References
Oph-A1	3,4-Dioxygenase oxygenase component large subunit	(Han 2008)
Oph-A2	3,4-Dioxygenase oxygenase component small	(Han 2008)
Oph-B	Phthalate dihydrodiol dehydrogenase	(Han 2008)
Oph-C	3,4-Dihydroxyphthalate decarboxylase	(Han 2008)
Oph-D	d-Galactonate transporter	(Han 2008)
Oph-H	Hemerythrin-like metal-binding protein	(Han 2008)
Oph-R	Transcriptional regulator, MarR family	(Han 2008)
FEH	Phthalic ester hydrolase	(Stanislauskienė et al. 2011)
HFDH	3,4-Dihydroxy-3,4-dihidrophthalate dehydrogenase	(Stanislauskienė et al. 2011)
FOXG	Phthalate dioxygenase large and small subunits	(Stanislauskienė et al. 2011)
FOXGS	Ferredoxin and reductase subunits	(Stanislauskienė et al. 2011)
FDK	3,4-Dihidroxyphthalate-2-decarboxylase	(Stanislauskienė et al. 2011)
Ptr A	Transporter ATPase	(Choi et al. 2005)
Tph-A2	Terephthalate 1,2-dioxygenase oxygenase component large subunit	(Han 2008)
Tph-A3	Terephthalate 1,2-dioxygenase oxygenase component small subunit	(Han 2008)
Tph-B	Terephthalate dihydrodiol dehydrogenase	(Han 2008)

**Table 3:** Phthalate-degrading genes identified from *Methylobacillus* sp. V29b genome

Amplicons	Primer name	Name of the gene	Amplicon size
84	Tph-B-F2,R2	Terephthalate dehydroxygenase	500 kb
85	Tph-B-F1,R1	Terephthalate dehydroxygenase	800 kb
86	Oph-A1	Phthalate dioxygenase	800 kb
87	Oph-D	Phthalate permease	1 kb
89	Oph-C	Phthalate decarboxylase	1 kb
93	Tph-B-F2,R3	Terephthalate dehydroxygenase	500 bp
95	Oph-H	Hemerythin-like metal-binding protein	200 bp
99	Oph-B	Phthalate dehydrogenase	500 bp

**Fig. 7:**
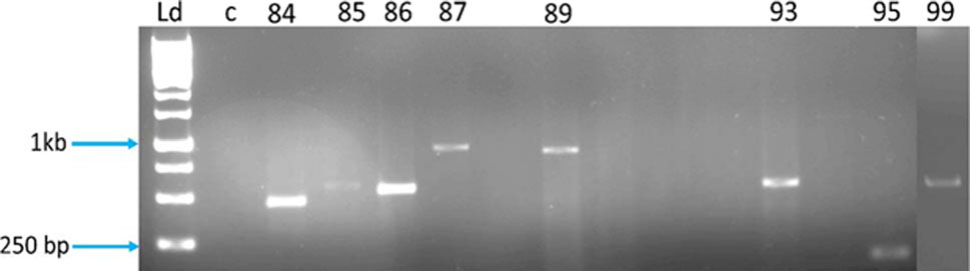
Phthalate esters-degrading genes amplified form the genome of *Methylobacillus* sp. V29b. Ld-ladder, c-control, 84, 85 and 93-Tph-B, 86-oph-A1, 87-oph-D, 89-oph-C, 95-oph-H, an 99-oph-B

Biodegradation of phthalate esters is initiated by their transport inside the cell by phthalate permease (oph-D) which induces phthalate 4,5 dioxygenase. They belong to major facilitator superfamily with 12 hydrophobic membrane-spanning helices. Phthalate permeases are reported as transport enzymes (Eaton 2001; Keyser et al. 1976). Permeases from *P. putida* NMH102-2 and *B. cepacia* ATCC 17616 found similarity with anion:cation symporter family (Chang and Zylstra 1999). Permeases have reported multiple genes and have catabolic operons (Chang and Zylstra 1999; Eaton 2001; Sasoh et al. 2006; Wang et al. 1995b). Phthalate dioxygenase (oph-A1) catalyzes the incorporation of two hydroxyl groups on the phthalate ring to yield phthalate dihydrodiols (Eaton 2001; Keyser et al. 1976). Primer oph-A1 was used to amplify the enzyme called phthalate dioxygenase reductase (PDR). PDR is an iron sulfur flavoprotein which utilizes flavin mononucleotide (FMN) to accomplish electron transfer from reduced nicotinamide adenine nucleotide (NADH) to the one-electron acceptor, [2Fe-2S] (Correll et al. 1992). Phthalate-4,5 dioxygenase action produces *cis*-4,5-dihydroxy-4,5-dihydrophthalate, which is dehydrogenated by *cis*-phthalate dihydrodiol dehydrogenase (oph-B) to 4,5-dihydroxyphthalate. Decarboxylation of 4,5-dihydroxyphthalate by 4,5-dihydroxyphthalate decarboxylase (oph-C) produces 3,4-dihydroxybenzoate, also known as protocatechuate (Batie et al. 1987; Chang and Zylstra 1998; 1999; Pujar and Ribbons 1985). Protocatechuate is one of the important intermediates in various pathways including phthalates (Eaton 2001). Protocatechuate then enters Krebs cycle after conversion to pyruvate and carbon dioxide. Protocatechuate is also present in the benzoate degradation pathway (Choi et al. 2005). Oph-H codes for a protein known as hemerythrin-like metal-binding protein. Hemerythrins are non-heme oxygen-binding proteins and they bind oxygen with a di-iron centre (Stenkamp 1994). They occur in invertebrates *Sipunculida* (peanut worms), *Brachiopoda* (lamp shells) *Priapulida* (priapulid worms) and some *Annelida* (segmented worms, including leeches and polychaete worms) (Klippenstein et al. 1968; Loehr et al. 1978; Long et al. 1992). Hemerythrin-like proteins were reported in prokaryotes, specifically *Methylococcus capsulatus* and *Desulfovibrio Vulgaris*, and it was proposed that the oxygen-binding domain acted as an oxygen sensor (Xiong et al. 2000). Genes for hemerythrin-like metal-binding proteins were amplified using oph-H primer from the bacteria-degrading phthalate, isophthalate and terephthalate. They have been found in cluster with genes responsible for phthalate degradation, but their specific function in PAEs degradation is uncertain (Han 2008). Terephthalate metabolism is initiated by double hydroxylation at the position 1 and 2 of the ring by terephthalate 1,2 dioxygnenase (Tph-B) to produce 2-hydro-1,2-dihydroxy terephthalic acid (Choi et al. 2005; Schläfli et al. 1994). 2-Hydro-1,2-dihydroxy terephthalic acid is further metabolized to 3,4-dihydroxybenzoate (Vamsee-Krishna et al. 2006; Wang et al. 1995b). Figure 7 presents the amplified gene products for phthalate ester-degrading genes.

This study is the first to report the isolation and characterization of a Gram-negative bacterium form of the genus *Methylobacillus-*degrading DBP. Bacteria from the genus *Methylobacillus* are methylotrophs and have the ribulose monophosphate (RuMP) pathway for formaldehyde assimilation (Bratina et al. 1992; Doronina et al. 2004; Urakami and Komagata 1986). The isolated *Methylobacillus* sp. V29b not only grows on methanol, but is able to grow on other substrates, such as protocatechuate, monobutyl phthalate, diethyl phthalate, benzyl butyl phthalate, dioctyl phthalate and diisodecyl phthalate. DBP degradation was reported by *Sphingmonas* sp. DK4 (5 mg/l) (Chang et al. 2004), *Pseudomonas fluorescens* B-1(2.5 & 10) (Xu et al. 2005), *Acinetobacter lwoffii* (1000 mg/l) (Hashizume et al. 2002), *Corynbacterium nitilophius* G11 (100 mg/l), *R. rhodochrous* G2, G7 (100 mg/l) (Chao et al. 2006) and *R. Coprophilus* G5, G9 (100 mg/l). *Methylobacillus* sp. V29b was able to degrade 1997 mg/l DBP, which is very high reported till so far. Degradation kinetics of organic pollutants were explained by first-order equations (Lu et al. 2009; Xu et al. 2005; Zeng et al. 2004), second-order equation (Yan et al. 1995), modified Gompertz model (Jin et al. 2012) and Haldane model (Wang et al. 1998). *Methylobacillus* sp. V29b demonstrated DBP degradation by the Monod model for growth inhibition, which is a new perspective to present the degradation of pollutants. While a majority of the studies are concentrated on the degradation of PAEs in minimal media (Chao et al. 2006; Fang et al. 2010; Wu et al. 2010a; Wu et al. 2010b), this study focuses on the degradation of DBP in contaminated samples collected from landfill sites. Landfill sites are composed of wastes from domestic, medical, pharmaceutical and industrial sources containing a variety of plastic items and PAEs. Therefore, it was selected for DBP degradation studies. It was an efficient degrader of DBP in PAEs-contaminated sample; therefore, it can be considered as a potential candidate for bioremediation of the sites contaminated with pollutants.

## References

[CR1] Anthony C (1982) Biochemistry of methylotrophs. Academic Press, Dublin

[CR2] Batie CJ, LaHaie E, Ballou D (1987). Purification and characterization of phthalate oxygenase and phthalate oxygenase reductase from Pseudomonas cepacia. J Biolog Chem.

[CR3] Benckiser G, Ottow J (1982). Metabolism of the plasticizer di-n-butylphthalate by Pseudomonas pseudoalcaligenes under anaerobic conditions, with nitrate as the only electron acceptor. Appl Environ Microbiol.

[CR4] Bratbak G, Dundas I (1984). Bacterial dry matter content and biomass estimations. Appl Environ Microbiol.

[CR105] Bratina BJ, Brusseau  GA, Hanson  RS (1992). Use of 16S rRNA analysis to investigate phylogeny of methylotrophic bacteria. Int J Syst Evol Microbiol.

[CR5] Cartwright CD, Owen SA, Thompson IP, Burns RG (2000). Biodegradation of diethyl phthalate in soil by a novel pathway. FEMS Microbiol Lett.

[CR6] Chang H-K, Zylstra GJ (1998). Novel organization of the genes for phthalate degradation from Burkholderia cepacia DBO1. J Bacteriol.

[CR7] Chang H-K, Zylstra GJ (1999). Characterization of the phthalate permease OphD from Burkholderia cepacia ATCC 17616. J Bacteriol.

[CR8] Chang B, Yang C, Cheng C, Yuan S (2004). Biodegradation of phthalate esters by two bacteria strains. Chemosphere.

[CR9] Chao W, Lin C, Shiung I, Kuo Y (2006). Degradation of di-butyl-phthalate by soil bacteria. Chemosphere.

[CR10] Chen J-a (2007). Degradation of environmental endocrine disruptor di-2-ethylhexyl phthalate by a newly discovered bacterium, *Microbacterium* sp. strain CQ0110Y. Appl Microbiol Biotechnol.

[CR11] Cheung JK, Lam RK, Shi M, Gu J-D (2007). Environmental fate of endocrine-disrupting dimethyl phthalate esters (DMPE) under sulfate-reducing condition. Sci Total Environ.

[CR12] Choi KY, Kim D, Sul WJ, Chae J-C, Zylstra GJ, Kim YM, Kim E (2005). Molecular and biochemical analysis of phthalate and terephthalate degradation by *Rhodococcus* sp. strain DK17. FEMS Microbiol Lett.

[CR13] Colborn T, vom Saal FS, Soto AM (1993). Developmental effects of endocrine-disrupting chemicals in wildlife and humans. Environ Health Perspect.

[CR14] Correll C, Batie C, Ballou D, Ludwig M (1992). Phthalate dioxygenase reductase: a modular structure for electron transfer from pyridine nucleotides to [2Fe-2S]. Science.

[CR73] Council of the European Union (1993) Council Regulation (EEC) No 793/93 of 23 March 1993 on the evaluation and control of the risks of existing substances. European Union, Copenhagen

[CR15] Dai G, Cui L, Song L, Cheng J, Zhong Y, Zhao R, Wang X (2005). Bladder epithelial cell proliferation of rats induced by terephthalic acid-calculi. Food Chem Toxicol.

[CR16] Dai G (2005). Induction of bladder lesion by terephthalic acid and its mechanism. Biomed Environ Sci.

[CR17] David RM, Moore MR, Cifone MA, Finney DC, Guest D (1999). Chronic peroxisome proliferation and hepatomegaly associated with the hepatocellular tumorigenesis of di (2-ethylhexyl) phthalate and the effects of recovery. Toxicol Sci.

[CR18] Doronina NV, Trotsenko YA, Kolganova TV, Tourova TP, Salkinoja-Salonen MS (2004). *Methylobacillus pratensis* sp. nov., a novel non-pigmented, aerobic, obligately methylotrophic bacterium isolated from meadow grass. Int J Syst Evol Microbiol.

[CR19] Eaton RW (2001). Plasmid-encoded phthalate catabolic pathway in Arthrobacter keyseri 12B. J Bacteriol.

[CR20] Eaton RW, Ribbons DW (1982). Metabolism of dimethylphthalate by *Micrococcus* sp. strain 12B. J Bacteriol.

[CR21] Engelhardt G, Wallnöfer PR (1978). Metabolism of di-and mono-*n*-butyl phthalate by soil bacteria. Appl Environ Microbiol.

[CR22] Fang C-R, Yao J, Zheng Y-G, Jiang C-J, Hu L-F, Wu Y-Y, Shen D-S (2010). Dibutyl phthalate degradation by *Enterobacter* sp. T5 isolated from municipal solid waste in landfill bioreactor. Int Biodeterior Biodegrad.

[CR23] Feiler H (1980) Fate of priority pollutants in publicly owned treatment works. In: Fate of priority pollutants in publicly owned treatment works. EPA

[CR24] Felsenstein J (1981). Evolutionary trees from DNA sequences: a maximum likelihood approach. J Mol Evol.

[CR25] Fisher JS (2004). Environmental anti-androgens and male reproductive health: focus on phthalates and testicular dysgenesis syndrome. Reproduction.

[CR26] Gesler RM (1973). Toxicology of di-2-ethylhexyl phthalate and other phthalic acid ester plasticizers. Environ Health Perspect.

[CR27] Gray LE, Ostby J, Furr J, Price M, Veeramachaneni DR, Parks L (2000). Perinatal exposure to the phthalates DEHP, BBP, and DINP, but not DEP, DMP, or DOTP, alters sexual differentiation of the male rat. Toxicol Sci.

[CR28] Gross FC, Colony JA (1973). The ubiquitous nature and objectionable characteristics of phthalate esters in aerospace technology. Environ Health Perspect.

[CR29] Gu J, Li J, Wang Y (2004) Degradation of the endocrine-disrupting dimethyl phthalate ester isomers by aerobic microorganisms isolated from mangrove sediment. In: 4th World Water Congress. September 20–24, 2004. Marrakesh, Morocco, p 9

[CR30] Han R (2008) Phthalate biodegradation: gene organization, regulation and detection. ProQuest

[CR31] Hashizume K, Nanya J, Toda C, Yasui T, Nagano H, Kojima N (2002). Phthalate esters detected in various water samples and biodegradation of the phthalates by microbes isolated from river water. Biol Pharm Bull.

[CR32] Hauser R, Meeker J, Singh N, Silva M, Ryan L, Duty S, Calafat A (2007). DNA damage in human sperm is related to urinary levels of phthalate monoester and oxidative metabolites. Human Reprod.

[CR33] Huang P-C, Tien C-J, Sun Y-M, Hsieh C-Y, Lee C-C (2008). Occurrence of phthalates in sediment and biota: relationship to aquatic factors and the biota-sediment accumulation factor. Chemosphere.

[CR34] Ito R (2005). Reducing the migration of di-2-ethylhexyl phthalate from polyvinyl chloride medical devices. Int J Pharm.

[CR35] Jaeger RJ, Rubin RJ (1970). Plasticizers from plastic devices: extraction, metabolism, and accumulation by biological systems. Science.

[CR36] Jiao Y (2013). Identification and characterization of a cold-active phthalate esters hydrolase by screening a metagenomic library derived from biofilms of a wastewater treatment plant. PloS One.

[CR37] Jin D-C, Liang R-X, Dai Q-Y, Zhang R-Y, Wu X-L, Chao W-L (2010). Biodegradation of di-n-butyl phthalate by *Rhodococcus* sp. JDC-11 and molecular detection of 3, 4-phthalate dioxygenase gene. J Microbiol Biotechnol.

[CR38] Jin D (2012). Biodegradation of di-n-butyl phthalate by an isolated *Gordonia* sp. strain QH-11: genetic identification and degradation kinetics. J Hazard Mater.

[CR39] Jobling S, Reynolds T, White R, Parker MG, Sumpter JP (1995). A variety of environmentally persistent chemicals, including some phthalate plasticizers, are weakly estrogenic. Environ Health Perspect.

[CR40] Kaun-Yu L, Fu-Wei T, Chia-Jung W, Pei-Shan L (2004). Suppression by phthalates of the calcium signaling of human nicotinic acetylcholine receptors in human neuroblastoma SH-SY5Y cells. Toxicology.

[CR41] Keith L, Telliard W (1979). ES&T special report: priority pollutants: Ia perspective view. Environ Sci Technol.

[CR42] Keyser P, Pujar BG, Eaton RW, Ribbons DW (1976). Biodegradation of the phthalates and their esters by bacteria. Environ Health Perspect.

[CR43] Klippenstein GL, Hollerman JW, Klotz IM (1968). Primary structure of Golfingia gouldii hemerythrin. Order of peptides in fragments produced by tryptic digestion of succinylated hemerythrin. Complete amino acid sequence. Biochemistry.

[CR44] Krauskopf L (1973). Studies on the toxicity of phthalates via ingestion. Environ Health Perspect.

[CR45] Li J, Gu JD (2007). Complete degradation of dimethyl isophthalate requires the biochemical cooperation between *Klebsiella oxytoca* Sc and *Methylobacterium mesophilicum* Sr isolated from wetland sediment. Sci Total Environ.

[CR46] Li L-H, Jester WF, Orth JM (1998). Effects of relatively low levels of mono-(2-ethylhexyl) phthalate on cocultured sertoli cells and gonocytes from neonatal rats. Toxicol Appl Pharmacol.

[CR47] Li J, Gu J-D, Yao J-h (2005). Degradation of dimethyl terephthalate by *Pasteurella multocida* Sa and *Sphingomonas paucimobilis* Sy isolated from mangrove sediment. Int Biodeterior Biodegrad.

[CR48] Liang D-W, Zhang T, Fang HH, He J (2008). Phthalates biodegradation in the environment. Appl Microbiol Biotechnol.

[CR49] Lidstrom ME (2006) Aerobic methylotrophic prokaryotes. In: The prokaryotes. Springer, New York, pp 618–634

[CR50] Loehr J, Lammers P, Brimhall B, Hermodson M (1978). Amino acid sequence of hemerythrin from Themiste dyscritum. J Biol Chem.

[CR51] Long RC, Zhang J-H, Kurtz DM, Negri A, Tedeschi G, Bonomi F (1992). Myohemerythrin from the sipunculid, *Phascolopsis gouldii*: purification, properties and amino acid sequence. Biochimica et Biophysica Acta (BBA)-Protein Struct Mol Enzymol.

[CR52] Lovekamp-Swan T, Davis BJ (2003). Mechanisms of phthalate ester toxicity in the female reproductive system. Environ Health Perspect.

[CR53] Lu Y, Tang F, Wang Y, Zhao J, Zeng X, Luo Q, Wang L (2009). Biodegradation of dimethyl phthalate, diethyl phthalate and di-n-butyl phthalate by *Rhodococcus* sp. L4 isolated from activated sludge. J Hazard Mater.

[CR54] Marcel YL (1973). Determination of di-2-ethylhexyl phthalate levels in human blood plasma and cryoprecipitates. Environ Health Perspect.

[CR55] Moore NP (2000). The oestrogenic potential of the phthalate esters. Reprod Toxicol.

[CR56] Park YN, Choi MS, Rehman SU, Gye MC, Yoo HH (2016). Simultaneous GC-MS determination of seven phthalates in total and migrated portions of paper cups. Environ Sci Pollut Res.

[CR57] Piersma AH, Verhoef A, te Biesebeek J, Pieters MN, Slob W (2000). Developmental toxicity of butyl benzyl phthalate in the rat using a multiple dose study design. Reprod Toxicol.

[CR58] Pujar B, Ribbons D (1985). Phthalate metabolism in Pseudomonas fluorescens PHK: purification and properties of 4, 5-dihydroxyphthalate decarboxylase. Appl Environ Microbiol.

[CR59] Rothera AC (1908). Note on the sodium nitro-prusside reaction for acetone. J Physiol.

[CR60] Sasoh M, Masai E, Ishibashi S, Hara H, Kamimura N, Miyauchi K, Fukuda M (2006). Characterization of the terephthalate degradation genes of *Comamonas* sp. strain E6. Appl Environ Microbiol.

[CR61] Schläfli HR, Weiss MA, Leisinger T, Cook AM (1994). Terephthalate 1, 2-dioxygenase system from *Comamonas testosteroni* T-2: purification and some properties of the oxygenase component. J Bacteriol.

[CR62] Schwarzbauer J, Heim S, Brinker S, Littke R (2002). Occurrence and alteration of organic contaminants in seepage and leakage water from a waste deposit landfill. Water Res.

[CR63] Shuler ML, Kargi F (2002). Bioprocess engineering.

[CR64] Smith KCACWO (1955). The scanning electron microscope and its fields of application Br. J Appl Phys.

[CR65] Stanislauskienė R, Rudenkov M, Karvelis L, Gasparavičiūtė R, Meškienė R, Časaitė V, Meškys R (2011) Analysis of phthalate degradation operon from *Arthrobacter* sp. 68b biologija 57

[CR66] Staples CA, Adams WJ, Parkerton TF, Gorsuch JW, Biddinger GR, Reinert KH (1997). Aquatic toxicity of eighteen phthalate esters. Environ Toxicol Chem.

[CR67] Stenkamp RE (1994). Dioxygen and hemerythrin. Chem Rev.

[CR68] Tamura K, Peterson D, Peterson N, Stecher G, Nei M, Kumar S (2011). MEGA5: molecular evolutionary genetics analysis using maximum likelihood, evolutionary distance, and maximum parsimony methods. Mol Biol Evol.

[CR69] Teil MJ, Blanchard M, Chevreuil M (2006). Atmospheric fate of phthalate esters in an urban area (Paris-France). Sci Total Environ.

[CR70] Tepper LB (1973). Phthalic acid esters—an overview. Environ Health Perspect.

[CR71] Thompson JD, Higgins DG, Gibson TJ (1994). CLUSTAL W: improving the sensitivity of progressive multiple sequence alignment through sequence weighting, position-specific gap penalties and weight matrix choice. Nucleic Acids Res.

[CR72] Thuren A (1986). Determination of phthalates in aquatic environments. Bull Environ Contam Toxicol.

[CR74] Urakami T, Komagata K (1986). Emendation of Methylobacillus Yordy and Weaver 1977, a genus for methanol-utilizing bacteria. Int J Syst Evol Microbiol.

[CR75] Vamsee-Krishna C, Phale P (2008). Bacterial degradation of phthalate isomers and their esters. Indian J Microbiol.

[CR76] Vamsee-Krishna C, Mohan Y, Phale PS (2006). Biodegradation of Phthalate Isomers by Pseudomonas aeruginosa PP4, *Pseudomonas* sp. PPD and Acinetobacter lwoffii ISP4. Appl Microbiol Biotechnol.

[CR77] Vega D, Bastide J (2003). Dimethylphthalate hydrolysis by specific microbial esterase. Chemosphere.

[CR78] Vikelsøe J, Thomsen M, Carlsen L (2002). Phthalates and nonylphenols in profiles of differently dressed soils. Sci Total Environ.

[CR79] Vos P et al. (2011) Bergey’s manual of systematic bacteriology: Volume 3: The Firmicutes, vol 3. Springer, Germany

[CR80] Wang YP, Gu J-D (2006). Degradability of dimethyl terephthalate by Variovorax paradoxus T4 and Sphingomonas yanoikuyae DOS01 isolated from deep-ocean sediments. Ecotoxicology.

[CR81] Wang J, Liu P, Qian Y (1995). Microbial degradation of di-n-butyl phthalate. Chemosphere.

[CR82] Wang YZ, Zhou Y, Zylstra GJ (1995). Molecular analysis of isophthalate and terephthalate degradation by *Comamonas testosteroni* YZW-D. Environ Health Perspect.

[CR83] Wang J, Liu P, Shi H, Qian Y (1998). Kinetics of biodegradation of Di-*n*-butyl phthalate in continuous culture system. Chemosphere.

[CR84] Wang Y, Fan Y, Gu J-D (2003). Aerobic degradation of phthalic acid by *Comamonas acidovoran* Fy-1 and dimethyl phthalate ester by two reconstituted consortia from sewage sludge at high concentrations. World J Microbiol Biotechnol.

[CR85] Wang Y, Fan Y, Gu J-D (2003). Microbial degradation of the endocrine-disrupting chemicals phthalic acid and dimethyl phthalate ester under aerobic conditions. Bull Environ Contam Toxicol.

[CR86] Wang Y, Fan Y, Gu J-D (2004). Dimethyl phthalate ester degradation by two planktonic and immobilized bacterial consortia. Int Biodeterior Biodegrad.

[CR87] Wang F, Xia X, Sha Y (2008). Distribution of phthalic acid esters in Wuhan section of the Yangtze River. China J Hazard Mater.

[CR88] Weisburg WG, Barns SM, Pelletier DA, Lane DJ (1991). 16S ribosomal DNA amplification for phylogenetic study. J Bacteriol.

[CR89] Wensing M, Uhde E, Salthammer T (2005). Plastics additives in the indoor environment—flame retardants and plasticizers. Sci Total Environ.

[CR90] Wilkinson CF, Lamb JC (1999). The potential health effects of phthalate esters in children’s toys: a review and risk assessment. Regul Toxicol Pharmacol.

[CR91] Wu X-l, Wang Y-y, Liang R-x, Dai Q-y, Chao W-l (2010). Degradation of di-*n*-butyl phthalate by newly isolated *Ochrobactrum* sp. Bull Environ Contam Toxicol.

[CR92] Wu X, Liang R, Dai Q, Jin D, Wang Y, Chao W (2010). Complete degradation of di-n-octyl phthalate by biochemical cooperation between *Gordonia* sp. strain JDC-2 and *Arthrobacter* sp. strain JDC-32 isolated from activated sludge. J Hazard Mater.

[CR93] Xiong J, Kurtz DM, Ai J, Sanders-Loehr J (2000). A hemerythrin-like domain in a bacterial chemotaxis protein. Biochemistry.

[CR94] XR Xu, Li HB, Gu JD (2006). Elucidation of n-butyl benzyl phthalate biodegradation using high-performance liquid chromatography and gas chromatography-mass spectrometry. Anal Bioanal Chem.

[CR95] Xu X-R, Li H-B, Gu J-D (2005). Biodegradation of an endocrine-disrupting chemical di-n-butyl phthalate ester by Pseudomonas fluorescens B-1. Int Biodeterior Biodegrad.

[CR96] Xu X-R, Li H-B, Gu J-D, Li X-Y (2007). Kinetics of *n*-butyl benzyl phthalate degradation by a pure bacterial culture from the mangrove sediment. J Hazard Mater.

[CR97] Yan H, Ye C, Yin C (1995). Kinetics of phthalate ester biodegradation by *Chlorella pyrenoidosa*. Environ Toxicol Chem.

[CR98] Yuan S, Liu C, Liao C, Chang B (2002). Occurrence and microbial degradation of phthalate esters in Taiwan river sediments. Chemosphere.

[CR99] Zeng F, Cui K, Li X, Fu J, Sheng G (2004). Biodegradation kinetics of phthalate esters by *Pseudomonas fluoresences* FS1. Process Biochem.

[CR100] Zheng Z, He P-J, Shao L-M, Lee D-J (2007). Phthalic acid esters in dissolved fractions of landfill leachates. Water Res.

[CR101] Zhu J, Phillips SP, Feng Y-L, Yang X (2006). Phthalate esters in human milk: concentration variations over a 6-month postpartum time. Environ Sci Technol.

